# Prospective multicenter validation of a next-generation sequencing panel using cytology specimens for lung cancer: cPANEL

**DOI:** 10.1186/s12885-025-14770-0

**Published:** 2025-10-09

**Authors:** Kei Morikawa, Yuta Takashima, Masahide Oki, Akifumi Tsuzuku, Shuji Murakami, Daisuke Minami, Shinji Fujii, Naofumi Shinagawa, Fumihiro Asano, Seiji Nakamura, Yoshiharu Sato, Yumi Ueda, Fumihiko Suzuki, Tomoyuki Yokose, Kenichiro Tanabe, Masamichi Mineshita

**Affiliations:** 1 Department of Respiratory Medicine, St. Marianna University School of Medicine, Kawasaki, Japan; 2 Department of Respiratory Medicine, Faculty of Medicine, Hokkaido University, Sapporo, Japan; 3 Department of Respiratory Medicine, NHO Nagoya Medical Center, Nagoya, Japan; 4 Department of Respiratory Medicine, Gifu Prefectural General Medical Center, Gifu, Japan; 5 Department of Thoracic Oncology, Kanagawa Cancer Center, Yokohama, Japan; 6 Department of Respiratory Medicine, Kawasaki Medical School, Kurashiki, Japan; 7 Department of Respiratory Medicine, Kumamoto Regional Medical Center, Kumamoto, Japan; 8 DNA Chip Research, Inc, Kawasaki, Japan; 9 Department of Pathology, Kanagawa Cancer Center, Yokohama, Japan; 10 Pathology and Bioregulation, St. Marianna University Graduate School of Medicine, Kawasaki, Japan

**Keywords:** Cytology specimen, Gene panel analysis, Next-generation sequencing, Non-small cell carcinoma, Variant allele frequency

## Abstract

**Background:**

There are no prospective studies to estimate whether cytology specimens can replace tissue samples using lung cancer gene panel analysis. We evaluated the success rate of gene panel testing and nucleic acid yield and quality when using cytology specimens for lung cancer over tissue specimens.

**Methods:**

In this prospective study, clinical cytology specimens collected via transbronchial brushing, needle aspiration washing, and pleural effusion were stored in a nucleic acid stabilizer. The primary endpoint was the superior success rate of gene analysis using cytology specimens over the conventional success rate using tissue specimens.

**Results:**

The full analysis set included 248 cases. The success rate for gene panel analysis using cytology specimens was 98.4% (95% confidence interval (CI), 95.9–99.6%) with a positive concordance rate of 97.3% (95% CI, 90.7–99.7%) by other companion diagnostic kits. The median value for nucleic acid yield and quality (DNA/RNA integrated number) of cytology specimens was 546.0/426.5 ng and 9.2/4.7 for DNA/RNA, respectively. The Pearson correlation coefficient of variant allele frequency between tissue formalin-fixed and paraffin-embedded (FFPE) sample and cytology specimens for mutant cases was 0.815. The ratio of double-stranded to total DNA showed that cytology specimens were of significantly higher quality than FFPE specimens.

**Conclusions:**

The success rate of cytology specimens in gene analysis was significantly higher than conventional data. Because of the sufficient nucleic acid yield, high quality, and high correlation of mutant allele frequency compared to FFPE specimens, cytology specimens are suitable for panel testing as tissue substitutes.

**Trial registration:**

UMIN Registry UMIN000047215 (cPANEL trial). https://center6.umin.ac.jp/cgi-open-bin/ctr_e/ctr_view.cgi?recptno=R000053766.

**Supplementary Information:**

The online version contains supplementary material available at 10.1186/s12885-025-14770-0.

## Introduction

Personalized medicine, particularly molecularly targeted drugs, has considerably improved patient response rates and long-term prognosis [1, 2] This is particularly attributable to the expanded detectability of rare driver mutations at initial diagnosis beyond epidermal growth factor receptor (*EGFR*) and anaplastic lymphoma kinase (*ALK*).

The Oncomine Dx Target Test Multi-CDx system (ODxTT, Thermo Fisher Scientific, San Jose, CA, USA), approved by the FDA in 2017, is a class-leading next-generation sequencing (NGS) panel for non-small cell lung cancer (NSCLC) [3]. However, this system requires adequate amounts of malignant cells in tissue samples and meticulous sample handling, often leading to small sample sizes with compromised quality [3–5]. Previous global clinical trials and multicenter real-world data indicated that the panel had suboptimal success rates of 72.0–90.0% [6–13]. Moreover, the method requires increased time and effort for the collection of larger specimens and macro-dissection in specimen preparation [7, 8]. Therefore, more efficient procedures with higher success rates and rapid analytical turnover are urgently required.

Cytology specimens are commonly used in clinical practice since they require minimally invasive collection techniques and yield quick results [14–16]. However, their use in genetic panel testing is not widely endorsed in international guidelines, although occasionally used for a single-plex test as clinical practice [17–20]. Furthermore, cytology specimens are not applicable for comprehensive genome profile tests such as FoundationOne^ฏ^ CDx (Foundation Medicine, Cambridge, MA, USA) and MSK-IMPACT^ฏ^(Memorial Sloan Kettering Cancer Center, New York, NY, USA) [21, 22]. Although studies involving international collaboration have demonstrated high concordance in mutation detection and variant allele frequencies (VAF) across different NGS platforms using archival cytology samples [23, 24], these studies typically utilized established cell lines rather than prospective clinical methods [25].

Therefore, this study primarily aimed to confirm the success rate of panel tests using cytology samples, and secondarily aimed to compare the quantity and quality of nucleic acids yielded from cytology versus tissue samples to elucidate the value of cytology samples and their handling in gene panel tests. Both cytology and tissue samples should be triaged appropriately according to their intended use, but if panel testing cannot be performed due to insufficient tissue collection, patients’ treatment options will be limited. In this study, we used the Lung Cancer Compact Panel™ (LCCP), a highly sensitive targeted next-generation sequencing panel, to evaluate cytology-based testing. Our results may aid in accelerating the application of gene panel tests for genetic mutation searches, thereby enhancing the development and application of personalized treatments.

## Methods

### Trial design

The cPANEL prospective phase 3 multicenter trial evaluates the feasibility of performing gene panel tests using cytology specimens collected via brushing cytology, needle aspiration washing solution, and pleural effusion in clinical practice. The study was conducted in accordance with the Declaration of Helsinki and the study was approved by the Institutional Review Board of St. Marianna University School of Medicine, Kawasaki, Japan (approval number 5532). Written informed consent was obtained from all patients prior to study initialization. The study registered in the UMIN Registry (UMIN000047215). An independent data-monitoring committee reviewed the clinical data.

### Study participants

Eligible participants were adults aged ≥ 20 years who underwent cytopathological diagnosis for suspected primary lung cancer. Secondary registration excluded patients diagnosed with benign and metastatic lung cancer or whose paired cytology specimens showed no malignant cells.

### Diagnostic procedures

Bronchoscopic evaluations were conducted using endobronchial ultrasonography (EU-ME2; Olympus, Tokyo, Japan), either with or without the assistance of a guide sheath kit (Olympus). EBUS-guided transbronchial needle aspiration (TBNA) or endoscopic ultrasound-guided fine-needle aspiration was performed via a flexible fiberscope, typically involving two to three passes with a 22-gauge needle. For CT or ultrasound (US)-guided core needle biopsies, a semi-automatic aspiration system (Temno Evolution, Care Fusion Japan, Tokyo, Japan) equipped with a 20-gauge needle (length 11–15 cm) was used in three attempts. When feasible, an adequate volume of tissue was obtained for further analysis.

### Cytology and tissue specimen collection

**Fig. 1:**
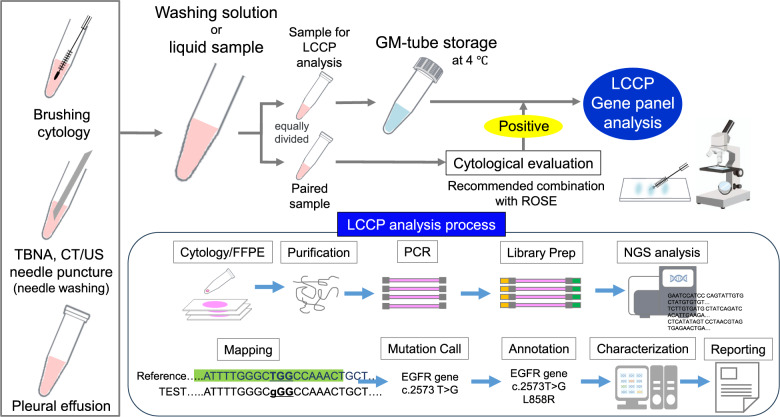
Cytology sample collection and LCCP analysis CT: computed tomography. LCCP: lung cancer compact panel. ROSE: rapid on-site cytologic evaluation. TBNA: transbronchial needle aspiration. US: ultrasound

Figure 1 outlines the process used for collecting cytology specimens. For transbronchial biopsies, lesion scraping was performed using a brush, with the collected material transferred onto a glass slide and agitated in 4 mL of normal saline two to three times. In the case of needle aspiration or biopsy, the core tissue was first harvested for histological analysis, followed by needle rinsing using approximately 1 mL of normal saline and air flushing (2–3 repetitions) to collect residual cells. The rinsing fluid was then equally split into two containers: one for routine sampling and the other for cytological examination (paired cytology samples). Patients with no malignant cells in the paired cytology samples were excluded from secondary registration. For pleural effusions, a minimum of 20 mL was collected, divided into two parts, centrifuged, and the cell pellets were preserved in containers for pathological assessment.

For tissue-based panel testing, formalin-fixed paraffin-embedded (FFPE) samples—including surgical specimens, core needle biopsies, and cell blocks—were processed and submitted according to institutional practice, based on the diagnostic pathology guidelines issued by the Japanese Society of Pathology. Histological assessment of tumor content (typically requiring ≥ 30%) was performed prior to submission, and macrodissection was applied when necessary. In contrast, cytology specimens—including bronchial brushing rinses, TBNA needle flush fluid, and pleural effusion samples—were preserved in a non-formalin, ammonium sulfate-based nucleic acid stabilizer (GM tube) and processed uniformly across participating sites using a standardized preprocessing protocol. At the time of the study, no clinical NGS panels had been implemented with sufficient analytical sensitivity for cytology samples, nor were there validated preservation methods for ensuring nucleic acid quality from liquid-based cytology; thus, submission of cytology specimens for routine panel testing was not feasible in conventional practice. This study was therefore designed to establish and validate a new workflow for cytology-based gene panel testing using ammonium sulfate–based preservation and to evaluate its analytical feasibility and clinical utility in a multicenter setting. Comparative analyses between FFPE and cytology samples were performed using matched samples derived from the same lesions whenever possible, and potential biases due to differences in institutional tissue processing were taken into consideration in the interpretation of results.

### Sample storage and transport conditions

Cytology specimens for the NGS panel were collected in a sample container (GM tube, GeneMetrics, Osaka, Japan) containing 2 mL of a nucleic acid stabilizer to inhibit DNase/RNase activity. After storage in GM tube, no centrifugation or freezing was required. The sample containers were refrigerated and shipped to the inspection agency (DNA Chip Research, Tokyo, Japan).

### Sample analysis

#### Sample purification

Sample preparation and nucleic acid purification were conducted using commercially available kits following the manufacturer’s instructions, as previously described [25]. Specifically, cytology specimens were processed using the Maxwell^®^ RSC Blood DNA and simplyRNA Cells Kits, while DNA and RNA from FFPE samples were extracted with the Maxwell^®^ RSC DNA FFPE and RNA FFPE Kits (Promega, Madison, WI, USA). Nucleic acid quantification was performed using a Qubit™ fluorometer with dsDNA HS Assay Kits and NanoDrop^®^ UV-spectrophotometry (Thermo Fisher Scientific, USA). DNA quality was evaluated using the Genomic DNA assay on a TapeStation system (Agilent) to determine the DNA Integrity Number (DIN). RNA quality was assessed using either the RNA HS assay on the TapeStation or the Bioanalyzer system (Agilent), providing RIN/eRIN values and DV200%. The ratio of double-stranded DNA to total DNA was also calculated to evaluate DNA integrity.

#### Library preparation and NGS sequencing

The Lung Cancer Compact panel™ (LCCP: DNA Chip Research) is an amplicon-based high-sensitivity NGS panel capable of measuring eight druggable genes in lung cancer, including *EGFR*, *BRAF*, *KRAS*, *ERBB2*, *ALK*, *ROS1*, *MET*, and *RET*. The LCCP was approved by the Ministry of Health, Labor, and Welfare as a multi-companion diagnostic kit (CDx) for lung cancer in Nobemver 2022 and is currently approved as a seven-gene CDx in Japan. The LCCP is characterized by highly sensitive mutation calls, with a limit of detection (LOD) of 0.14%, 0.20%, 0.48%, 0.24%, and 0.20% for driver mutations such as the *EGFR* exon-19 deletion, L858R, T790M, *BRAF* V600E, and *KRAS* G12C, respectively. Using purified nucleic acid, the LCCP assay along with library preparation, NGS sequencing (MiSeq; Illumina, San Diego, CA, USA), and data analysis were performed as previously described [26]. In brief, amplicon based multiplex PCR were performed to amplify targeting regions. As a design concept of the compact panel™, the sizes of target amplicon regions were optimized as narrow as possible to increase amplifiability of tumor-derived nucleic acids. Sequence libraries were constructed using the GenNext^®^ NGS Library Prep Kit (Toyobo) from purified PCR products. Sequence data was obtained using MiSeq (Illumina, CA, USA) for the constructed sequence library (2 × 150 bp paired-end mode).

### Paired cytology specimen diagnosis

Cytology specimen diagnosis was evaluated according to the World Health Organization’s (WHO) reporting system for lung cytopathology [27, 28] Among the five categories of lung cytopathological specimen types, atypical, suspicious for malignancy, and malignant diagnoses in paired specimens were considered for secondary registration, regardless of the tumor cell content. The cytological diagnoses and evaluations were confirmed by multiple pathologists and cytologists at each institution. Paired cytology samples from cases in which a genetic mutation was detected by the LCCP were sequentially collected for central evaluation.

### Pathological diagnosis and CDx

Histopathological diagnosis was performed according to the 2015 WHO Classification of Tumors of the Lung [29]. A medical insurance-approved genetic test was performed as CDx. When sufficient samples could be collected, the samples were preferentially evaluated using the ODxTT or an Amoy 9-in-1 kit (Amoy Diagnostics, Xiamen, China). For single gene searches, a Cobas^®^ EGFR mutation test was used to detect *EGFR* mutations; immunohistochemistry, Ventana OptiView ALK (D5F3; Roche Molecular Systems, Pleasanton, CA, USA) and Vysis^®^ ALK Break Apart FISH probe kit (Abbott Japan, Tokyo, Japan) were used to detect *ALK* mutations; and Archer^®^ MET (Invitae, San Francisco, CA, USA) was used to detect *MET* exon-14-skipping mutations. All other rare gene mutations were confirmed by ODxTT or Amoy detection.

### Concordance in variant allele frequencies (VAFs) between formalin-fixed paraffin-embedded (FFPE) tissue and cytology specimens

The VAF concordance between the cytology panel and FFPE tissue panel assays was assessed in patients with genetic mutations to clarify the reliability of the gene mutation allele frequency in cytology samples. Liquid pleural effusion samples were used as cell blocks for tissue substitutes. Macrodissection of tissue samples was not specified in the protocol, it was performed at the discretion of each institution when the tumor content was low. Four 10-µm-thick FFPE slides, two for DNA extraction and two for RNA extraction, were prepared per case. The VAF of the primary oncogenic mutation was selected as the best indicator of the tumor cell content for each sample.

#### Outcome assessments

The primary endpoint was to demonstrate the superiority of the LCCP when using cytology specimens, targeting a 90% success rate. This threshold was selected based on the upper bound of the 95% confidence interval (88.1%) observed in previous reports of multiple conventional panel tests using tissue samples (Supplementary eFigure 1).

A successful result was defined by the extraction of at least 10 ng of both DNA and RNA, along with sufficient NGS sequencing depth: ≥ 5,000 reads for the DNA diagnostic module, ≥ 2,000 reads for the DNA research module, and ≥ 300 reads for the internal reference gene HPRT1 in the RNA module. Secondary endpoints included mutation detection in eight key lung cancer-related genes (EGFR, BRAF, ALK, ROS1, MET, RET, KRAS, and HER2), the concordance rate between LCCP results and established companion diagnostics, and a reduction in test result turnaround time when using cytology-based LCCP. Exploratory outcomes involved comparing nucleic acid yields, DNA Integrity Number (DIN), and RNA Integrity Number (RIN) across cytology collection methods. Additionally, for mutation-positive cases, variant allele frequency (VAF) concordance between cytology and FFPE tissue samples was evaluated.

### Sample size

We set the expected value of the success rate in this trial at 95%, the threshold success rate at 90%, the one-sided significance level at 2.5%, and power at 80%, and calculated the sample size using an exact test of binomial proportion (upper one-sided test). If the sample size was ≥ 243, the power of the test would always be ≥ 80%. Therefore, 243 patients were required for secondary registration (power: 0.839). Considering patient withdrawal of consent, we set the required number of secondary registrations to 248.

### Statistical analysis

The Clopper and Pearson exact methods were used to calculate 95% CIs for binomial proportion testing. *p* < 0.05 was considered statistically significant. All analyses were performed using SAS version 9.4 (SAS Institute, Cary, NC, USA).

## Results

**Fig. 2:**
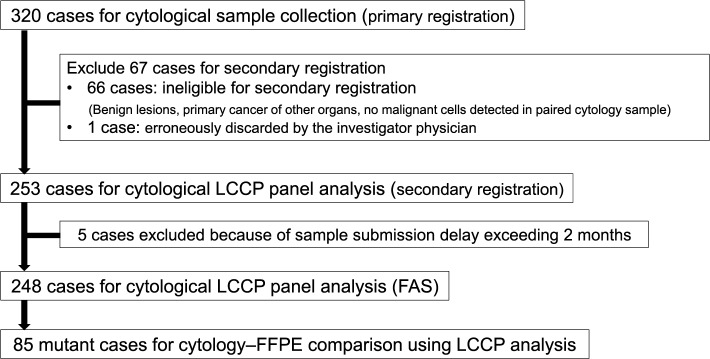
Flow diagram of case registration FAS: full analysis set. FFPE: formalin-fixed paraffin-embedded; LCCP: lung cancer compact panel

In total, 320 patients were enrolled in the primary registration between March 23, 2022, and March 1, 2023. We excluded 66 patients from the secondary registration, and one patient was erroneously discarded by the physician. Therefore, 253 patients were enrolled in the secondary registration, of which five were excluded because more than 2 months had elapsed between specimen collection and submission. Finally, 248 patients were included in the full analysis set (FAS, Fig. 2).

Baseline patient characteristics are summarized in Table 1. The median age was 70 years (range: 31–90), with 158 patients (63.7%) being male. Clinical stages I, II, III, IV, and unknown were observed in 33 (13.3%), 25 (10.1%), 57 (23.0%), 132 (53.2%), and 1 (0.4%) cases, respectively. Histological classifications included adenocarcinoma in 153 patients (61.7%), squamous cell carcinoma in 42 (16.9%), small cell carcinoma in 28 (11.3%), and non-specified carcinoma in 25 (10.1%). Cytology specimens were collected using various procedures: 133 (53.6%) via transbronchial brushing (TBB), 56 (22.6%) via TBNA, 32 (12.9%) via ultrasound- or CT-guided puncture, 20 (8.1%) from pleural effusion, and 7 (2.8%) through other approaches (Table 1).

**Table 1:** Patient characteristics and LCCP analysis

Patient characteristics	*n* = 248, case (%)	Pathological diagnosis	*n* = 248, case (%)
**Sex**		Adenocarcinoma	153 (61.7)
Male	158 (63.7)	Squamous cell carcinoma	42 (16.9)
Female	90 (36.3)	Small cell carcinoma	28 (11.3)
**Median age**, years (range)	70 (31–90)	Not otherwise specified, other	25 (10.1)
**Clinical stage**		**LCCP analysis success** (rate)	244 (98.4)
I	33 (13.3)	**LCCP mutation detection**	case
II	25 (10.1)	*EGFR*	59
III	57 (23.0)	*KRAS G12X*,* G13X*	24
IV	132 (53.2)	*KRAS G12C*	15
**Diagnostic procedure**		*ALK*	8
EBUS-TBB	133 (53.6)	*BRAF*	4
EBUS-TBNA, EUS-FNA	56 (22.6)	*MET ex14 skip.*	3
CT/US guided puncture	32 (12.9)	*RET*	3
Pleural effusion	20 (8.1)	*HER2*	2
Other	7 (2.8)	*ROS1*	1

*CT* Computed tomography, *EBUS-TBB* Endobronchial ultrasonography-guided transbronchial brushing, *EBUS-TBNA* Endobronchial ultrasonography-guided transbronchial needle aspiration, *EUS-FNA* Endoscopic ultrasound-guided fine needle aspiration, *LCCP L*ung cancer compact panel, *US* Ultrasound

The success rate of LCCP genetic alteration testing, which was the primary endpoint, for all eight genes using cytology specimens was 98.4% (95% CI: 95.9–99.6%, Table 1), showing superiority over the 90% success rate of conventional NGS panel tests (*p* < 0.001).

The positive concordance and predictive rates of the LCCP (secondary endpoint) were 97.4% (95% CI: 91.0–99.7%) and 93.8 (95% CI: 86.0–97.9%), respectively, for patients with genetic mutations using conventional CDx tests covered by public health insurance (Table S1).

**Fig. 3:**
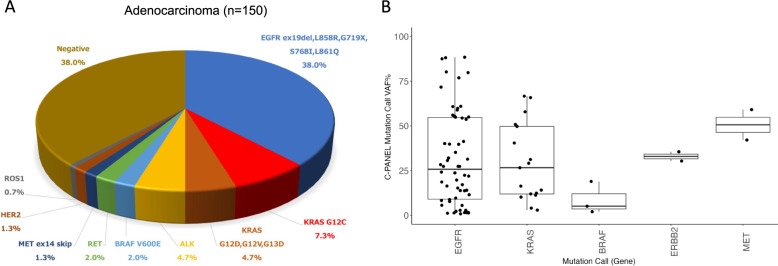
Pie chart of mutation calls detected by LCCP assay for adenocarcinoma (**A**) and each mutation VAF% (**B**) EBUS-TBB: endobronchial ultrasonography-guided transbronchial brushing. EBUS-TBNA: endobronchial ultrasonography-guided transbronchial needle aspiration. EUS-FNA: endoscopic ultrasound-guided fine needle aspiration. CT: computed tomography. US: ultrasound. VAF: variant allele frequency. FFPE: formalin-fixed paraffin-embedded

Among the 244 cases in the FAS in which genetic analysis was successful, driver gene mutations were detected in 103 (42.2%) using the LCCP (Table S2). In 150 cases of lung adenocarcinoma, the LCCP revealed driver gene mutations in 93 (62%) (Fig. 3A) There was no difference in mutation detection rate between nucleic acid yields of 10–100 ng (60.7%) and those of more than 100 ng (62.3%). The proportions of mutation VAF% for <5%, 5-<10%, 10-<20%, 20-<30%, 30-<40%, 40-<50%, 50%≦, were 15.9%, 8.5%, 19.5%, 12.2%, 11.0%, 6.1%, 26.8%, respectively (Fig. 3B).

The median turn-around time (TAT), defined in this study as the number of days from cytology sample collection to the reporting of results, was 12.0 days in the overall cohort (*n* = 248). This represents a revision from the original analysis, in which TAT was calculated from the date of specimen submission, we recalculated TAT using the sample collection date (day 0). Stratification by ROSE (rapid on-site cytologic evaluation) status revealed a significantly shorter TAT in the ROSE group (median: 10.0 days, interquartile range [IQR]: 9–14, *n* = 150) compared to the non-ROSE group (median: 15.0 days, IQR: 11–22, *n* = 98), with statistical significance confirmed by the Mann–Whitney U test (*p* <0.001). Supplementary Figure S2 visualizes the TAT distribution by ROSE status, clearly illustrating this benefit. Furthermore, ROSE implementation rates varied considerably across institutions: 93.3% at Kawasaki Medical School Hospital, 84.4% at Nagoya Medical Center, 51.1% at Hokkaido University Hospital, and 0.0% at both Gifu General Medical Center and Kumamoto Regional Medical Center. This inter-institutional variation likely contributed to differences in workflow speed and highlights the role of ROSE in enabling same-day specimen triage and shipping. In contrast to cytology samples, FFPE tissue specimens typically require multiple time-consuming steps, including formalin fixation, paraffin embedding, histopathological evaluation, and tumor content assessment, all of which delay genetic testing. Cytology-based panel testing avoids these delays, offering a clear time advantage.

The LCCP success rate was analyzed for each specimen collection method and was 99.2% (95% CI: 95.9–100.0%) for TBB; 96.4% (95% CI: 87.7–99.6%) for TBNA; 100% (95% CI: 89.1–100.0%) for CT- or US-guided puncture; and 100% (95% CI: 83.2–100.0%) for pleural effusion.

**Fig. 4:**
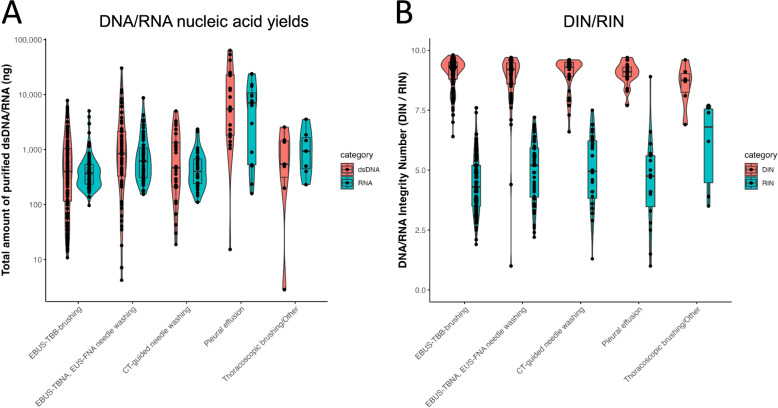
Nucleic acid yield (**A**) and quality (**B**) according to sample collection method DIN: DNA Integrity Number. RIN: RNA Integrity Number EBUS-TBB: endobronchial ultrasonography-guided transbronchial brushing. EBUS-TBNA: endobronchial ultrasonography-guided transbronchial needle aspiration. EUS-FNA: endoscopic ultrasound-guided fine needle aspiration. CT: computed tomography. US: ultrasound

**Fig. 5:**
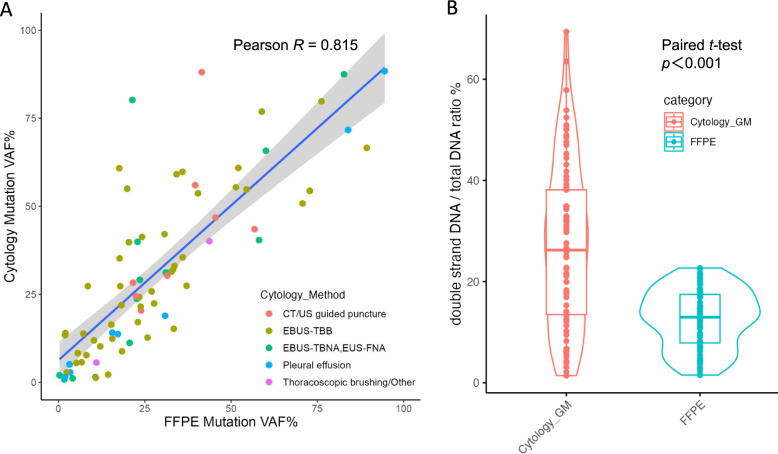
Correlations of VAF% (**A**) and purified DNA quality (**B**) between FFPE tissue and cytology samples VAF: variant allele frequency. FFPE: formalin-fixed paraffin-embedded

The median nucleic acid yield and DIN/RIN in the cytology specimens were 546.0/426.5 ng (Fig. 4A) and 9.2/4.7 for DNA/RNA, respectively (Fig. 4B, Table S3). Of 94 FFPE tissues, tumor content was recorded in 83 cases, with a median of 30% and a mean of 37% (range 2%−80%), all of which were successfully analyzed using LCCP. The Pearson correlation coefficient of VAFs between tissue FFPE samples and cytology specimens was 0.815 for the 85 cases where mutants were detected in cytology specimens (Fig. 5A, Table S4). The ratio for double-stranded DNA: total DNA was 27.1/12.8% for cytology: FFPE specimens, indicating the higher quality of cytology specimens compared to the FFPE specimens (paired *t*-test, *p* < 0.001; Fig. 5B, Table S5). Additionally, background noise during mutation analysis was significantly lower in cytology samples than in FFPE samples (Figure S3, Table S6).

## Discussion

This is the first multicenter study to prospectively evaluate the feasibility of an NGS lung cancer gene panel using cytology specimens. Our findings demonstrated a greater success rate for gene analysis using cytology specimens over conventional methods. Due to the adequate nucleic acid yield, high quality, and strong positive mutant allele frequency correlation with FFPE specimens, cytology specimens have the potential to replace tissue samples for detecting activating gene mutations [30]. 

The success rate of the LCCP using cytology specimens far surpassed that of conventional methods. Of 248 FAS cases, only four failed due to either defective *HER2* gene amplification or insufficient DNA yield, whereas RNA analysis showed no failures, regardless of RNA being more degradation-prone [31–33]. In the 150 lung adenocarcinoma cases, mutations were detected in 62% cases, which is highly consistent with other CDx methods [6, 9]. 

Sufficient nucleic acid yield and quality was confirmed regardless of the sample collection method. Moreover, the cytology specimens contained a significantly higher amount of double-stranded DNA compared to the FFPE samples collected during the same examination, suggesting higher-quality preservation. The cytology specimens are also less susceptible to external damage until nucleic acid extraction compared to FFPE specimens [34, 35] Notably, GM tube storage is highly versatile and useful for maintaining the quality of cytology specimens.

Our results revealed a strong correlation between the VAFs detected in cytology and tissue specimens. In contrast, in approximately half of the cases, a higher allele ratio was detected in the cytology specimens. Furthermore, our findings are consistent with the fact that cytological diagnosis has the advantage of producing faster results than histological diagnosis, thereby shortening the time between specimen collection and sample submission [36]. Importantly, in real-world clinical implementation of the LCCP following this study, the median TAT from specimen shipping to reporting has improved to 8–9 days. In institutions like St. Marianna University Hospital, where ROSE is routinely implemented, the sample collection date and shipping date were identical in most cases (median difference: 0 days), meaning that the actual TAT from collection to reporting is effectively also 8–9 days in practice. These findings underscore the clinical utility, operational speed, and scalability of cytology-based molecular diagnostics when combined with ROSE in real-world settings.

We performed a few additional analyses to investigate the discordance in results between the LCCP and CDx tests (Table S7). In the two cases where there was no positive match with the LCCP, both harbored *EGFR* exon-21 L858R point mutations. In the first case, the LCCP detected a VAF of 0.1%, below the LOD of 0.14%. In the second case, a procedural error occurred, in which 2 mL of the pleural effusion was directly injected into the GM tube without centrifugation. Of the five cases with discordant positive predictive values, minor allelic frequency mutations were detected by the LCCP in two cases, while one case had a co-mutation of *EGFR* Ex19del and *KRAS* G12C. The other three cases, including two cases with an *ALK* fusion gene and one with a *RET* fusion gene, were associated with either false positives or sub-minor clones. In discordant cases, the signal remained around the LOD, and a large deviation between tumor content and the estimated VAF was estimated by the pathologist.

This study had a few methodological limitations. First, this study did not perform a direct comparison but rather a verification of superiority based on a 90% success rate threshold calculated from pooled historical tissue panel data [7, 8, 10–12, 37]. Some recent studies have reported high diagnostic rates using other NGS procedures, however, they were based on retrospective analyses using archived samples with known gene mutation information [38]. Second, the LCCP gene panel test is limited to eight druggable driver mutations but the amount of nucleic acid sample (both DNA and RNA) necessary for testing is equivalent to that used for ODxTT (10 ng). However, LCCP has a much larger variant search than other NGS or PCR panels (Figure S4). LCCP was designed to expand measurable driver mutations and variants in the near future. Third, this study involved prospective case enrollment in clinical practice, the low number of rare fusion gene mutations was a methodological limitation. Finally, the integration of an automated assay system can significantly reduce sample processing time and increase the efficiency of genetic analysis. This potential advance is expected to further shorten TAT. Research is currently underway to use less invasive specimen collection techniques and to utilize cytology specimens for other solid tumors [39, 40].

This study was conducted as a multicenter prospective trial, and cytology specimens were collected and processed independently across seven participating institutions. To assess inter-institutional reproducibility, we compared the success rate of the LCCP assay by site. The results are summarized in Supplementary Table S8. All institutions demonstrated high success rates. The four failed cases were derived from different institutions, indicating no clustering of unsuccessful results. These findings support the robustness and consistency of cytology-based gene panel testing using the LCCP platform across diverse clinical practice settings.

In conclusion, our study demonstrated a high success rate for gene panel analysis using cytology specimens (98.4%). Based on the sufficient nucleic acid yield, high quality, and strong correlation in mutant allele frequency from FFPE specimens, we concluded that cytology specimens are suitable for gene panel testing as a substitute for tissue samples.

## Supplementary Information

Supplementary Material 1.

Supplementary Material 2.

Supplementary Material 3.

Supplementary Material 4.

Supplementary Material 5.

Supplementary Material 6.

Supplementary Material 7.

Supplementary Material 8.

Supplementary Material 9. Supplementary Figures.

## Data Availability

All data generated or analyzed during this study are included in this published article and its supplemental file.Additional raw data supporting the findings of this study are available from the corresponding author upon request.

## Abbreviations

FFPE: formalin-fixed paraffin-embedded
LCCP: Lung Cancer Compact panel
NGS: next-generation sequencing
NSCLC: Non-small cell lung cancer
ODxTT: Oncomine Dx Target Test Multi-CDx system
VAF: Variant allele frequencies
