# Supplementary material for: Prospective multicenter validation of a next-generation sequencing panel using cytology specimens for lung cancer: cPANEL
Source: BMC Cancer. 2025 Oct 9;25:1538. doi: 10.1186/s12885-025-14770-0 (PMC12512683; doi:10.1186/s12885-025-14770-0)
Supplement: Supplementary file 1 — Supplementary Material 1. [file 12885_2025_14770_MOESM1_ESM.docx]

**Supplement Table S1. Concordance of mutation call by CDx-tissue and LCCP-cytology.**

|  |  |  | **LCCP Cytology** | |  |  |  |  |
| --- | --- | --- | --- | --- | --- | --- | --- | --- |
|  |  | **gene** | **Positive** | **Negative** | **Sensitivity** | **Specificity** | **PPV** | **NPV** |
| **Tissue CDx DNA** | **Positive** | ***EGFR*** | 53 | 2 | 0.964 | - | 0.964 | - |
|  | **Negative** |  | 2 | 109 | - | 0.982 | - | 0.982 |
|  | **Positive** | ***BRAF V600E*** | 3 | 0 | 1.000 | - | 0.600 | - |
|  | **Negative** |  | 2 | 98 | - | 0.980 | - | 1.000 |
|  | **Positive** | ***KRAS G12C*** | 4 | 0 | 1.000 | - | 0.667 | - |
|  | **Negative** |  | 2 | 83 | - | 0.976 | - | 1.000 |
|  | **Positive** | ***HER2*** | 2 | 0 | 1.000 | - | 1.000 | - |
|  | **Negative** |  | 0 | 87 | - | 1.000 | - | 1.000 |
| **Tissue CDx RNA** | **Positive** | ***ALK*** | 6 | 0 | 1.000 | - | 0.750 | - |
|  | **Negative** |  | 2 | 102 | - | 0.981 | - | 1.000 |
|  | **Positive** | ***RET*** | 2 | 0 | 1.000 | - | 0.667 | - |
|  | **Negative** |  | 1 | 100 | - | 0.990 | - | 1.000 |
|  | **Positive** | ***MET*** | 2 | 0 | 1.000 | - | 1.000 | - |
|  | **Negative** |  | 0 | 87 | - | 1.000 | - | 1.000 |
|  | **Positive** | ***ROS1*** | 1 | 0 | 1.000 | - | 1.000 | - |
|  | **Negative** |  | 0 | 102 | - | 1.000 | - | 1.000 |
| **Tissue** | **Positive** | ***All 8***  ***genes*** | 73 | 2 | 0.973 | - | 0.890 | - |
| **CDx** | **Negative** |  | 9 | 768 | - | 0.988 | - | 0.997 |

CDx: companion diagnostic test. LCCP: lung cancer compact panel. PPV: positive predictive value. NPV: negative predictive value.
