# Supplementary figures and images for: Prospective multicenter validation of a next-generation sequencing panel using cytology specimens for lung cancer: cPANEL
Source: BMC Cancer. 2025 Oct 9;25:1538. doi: 10.1186/s12885-025-14770-0 (PMC12512683; doi:10.1186/s12885-025-14770-0)

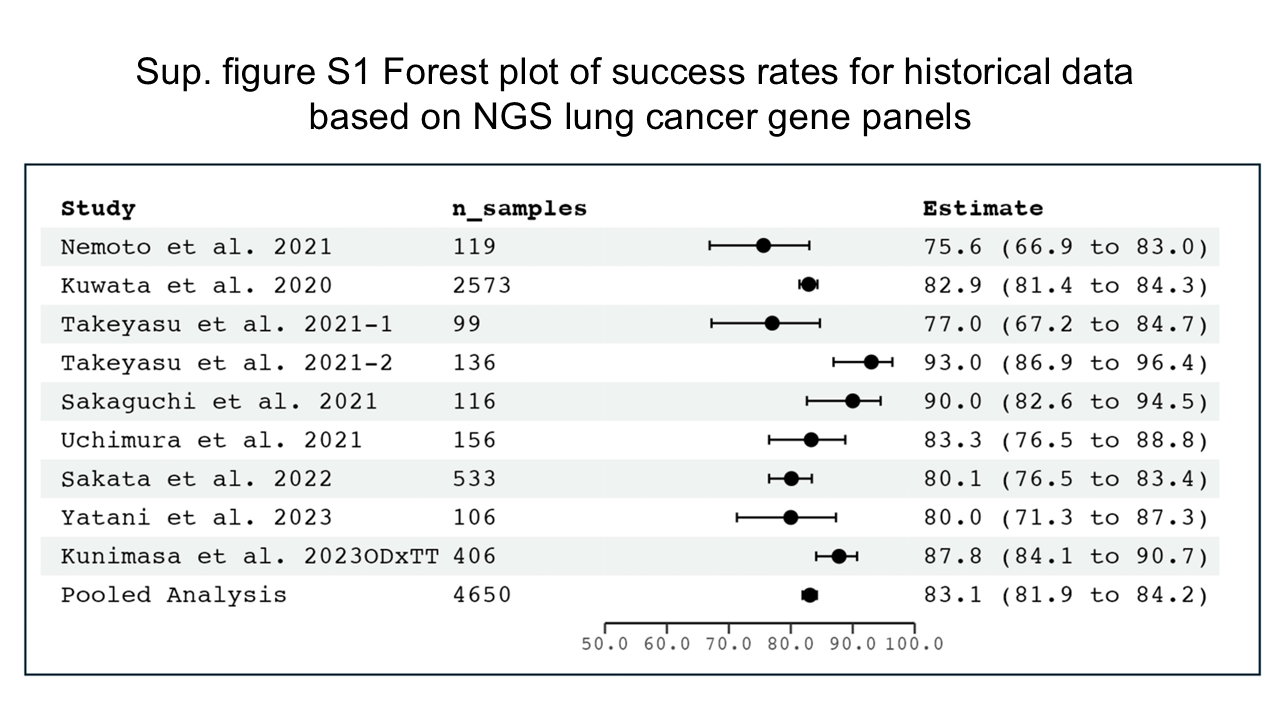

Supplement: Supplementary file 9 — Supplementary Material 9. Supplementary Figures. [file 12885_2025_14770_MOESM9_ESM.zip › 12885_2025_14770_MOESM9_ESM/Fig. S1.TIF]

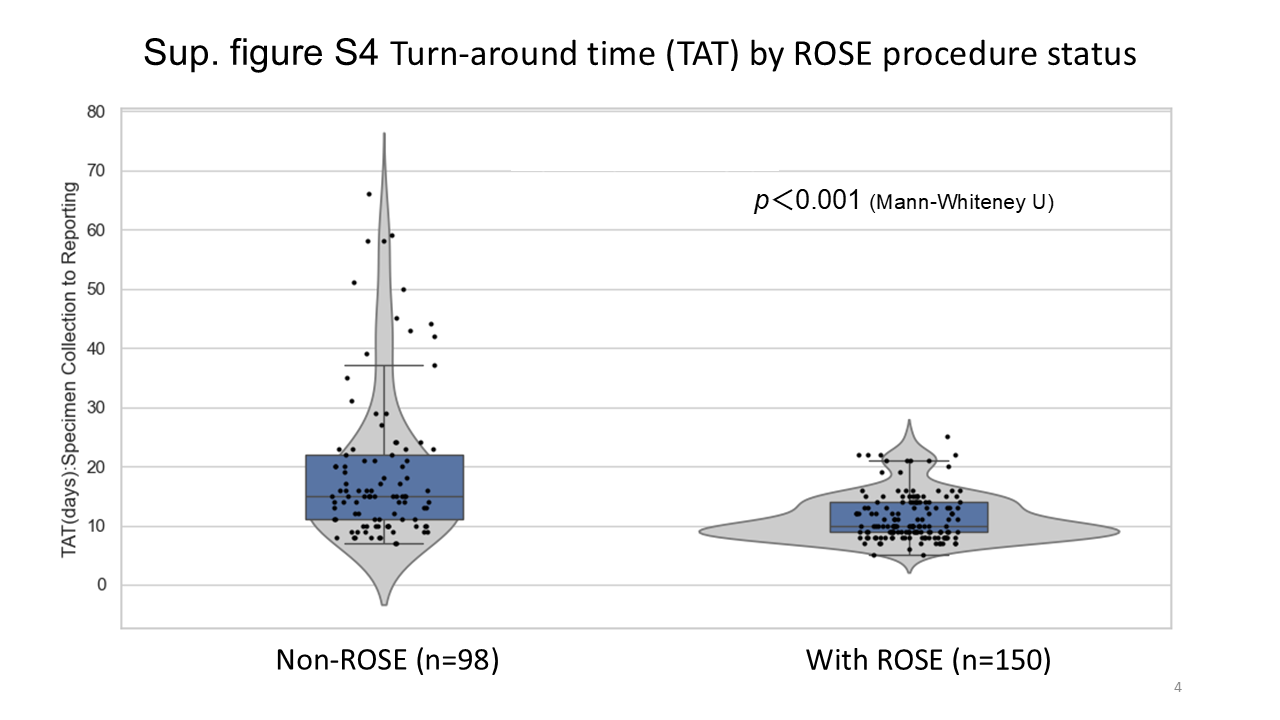

Supplement: Supplementary file 9 — Supplementary Material 9. Supplementary Figures. [file 12885_2025_14770_MOESM9_ESM.zip › 12885_2025_14770_MOESM9_ESM/Fig. S2.tif]

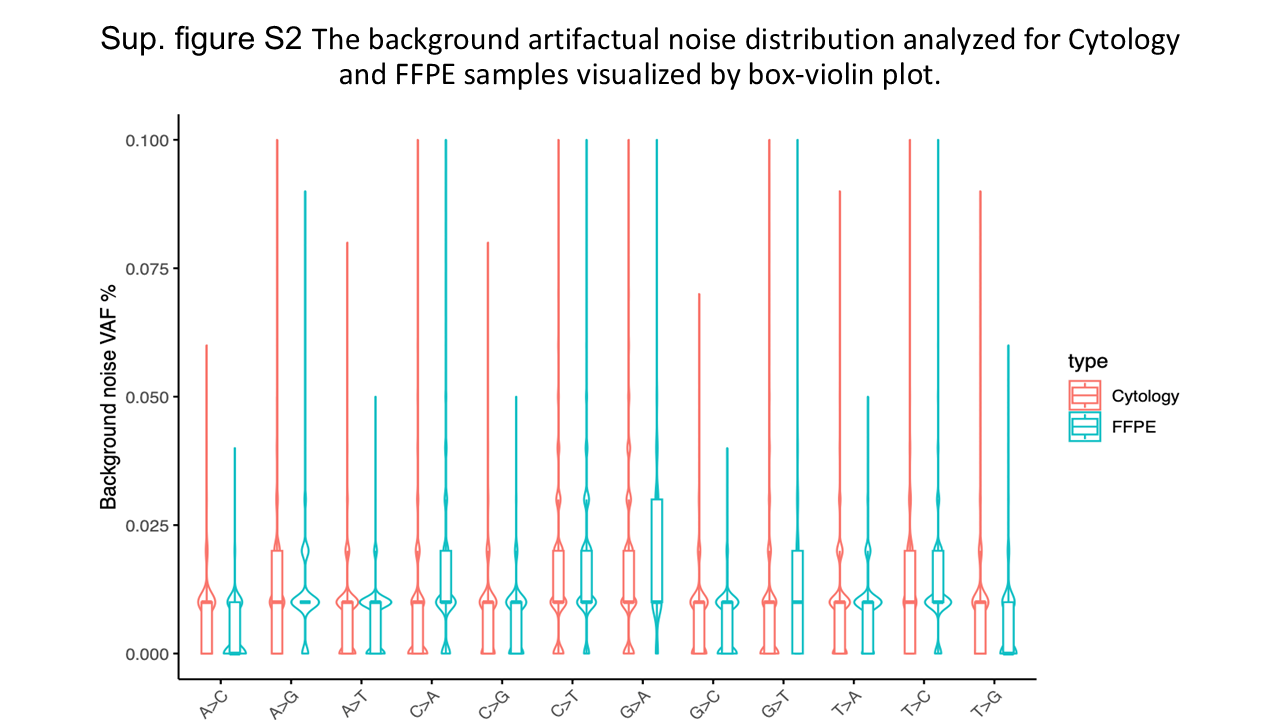

Supplement: Supplementary file 9 — Supplementary Material 9. Supplementary Figures. [file 12885_2025_14770_MOESM9_ESM.zip › 12885_2025_14770_MOESM9_ESM/Fig. S3.TIF]

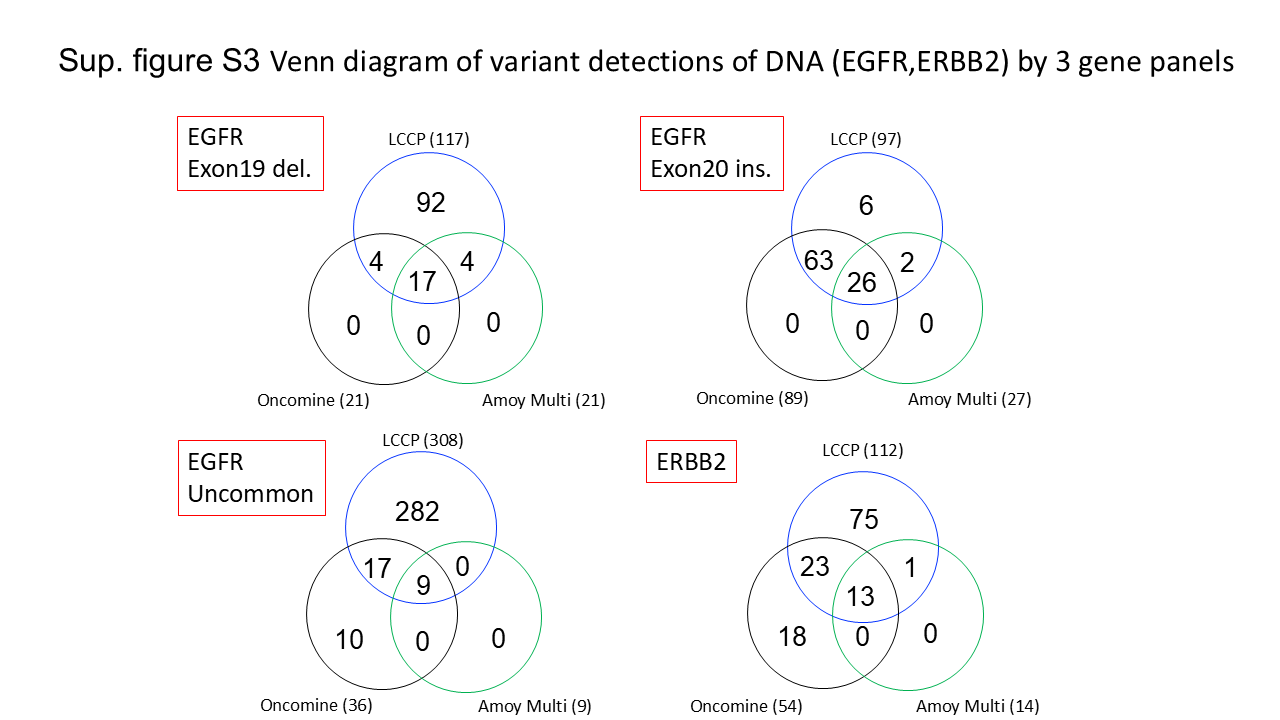

Supplement: Supplementary file 9 — Supplementary Material 9. Supplementary Figures. [file 12885_2025_14770_MOESM9_ESM.zip › 12885_2025_14770_MOESM9_ESM/Fig. S4.TIF]
